# Functional analysis of the secondary HIV-1 capsid binding site in the host protein cyclophilin A

**DOI:** 10.1186/s12977-019-0471-4

**Published:** 2019-04-04

**Authors:** Wang Peng, Jiong Shi, Chantal L. Márquez, Derrick Lau, James Walsh, K. M. Rifat Faysal, Chang H. Byeon, In-Ja L. Byeon, Christopher Aiken, Till Böcking

**Affiliations:** 1 EMBL Australia Node in Single Molecule Science and ARC Centre of Excellence in Advanced Molecular Imaging, School of Medical Sciences, UNSW, Sydney, NSW 2052 Australia; 2 Department of Pathology, Microbiology and Immunology, Vanderbilt University Medical Center, Nashville, TN USA; 3 Pittsburgh Center for HIV Protein Interactions, University of Pittsburgh School of Medicine, Pittsburgh, PA USA; 4 Department of Structural Biology, University of Pittsburgh School of Medicine, Pittsburgh, PA USA

**Keywords:** HIV-1, Capsid, Cyclophilin A, Non-canonical binding site

## Abstract

**Background:**

Efficient HIV-1 replication depends on interaction of the viral capsid with the host protein cyclophilin A (CypA). CypA, a peptidylprolyl isomerase, binds to an exposed loop in the viral CA protein via the enzyme’s active site. Recent structural analysis of CypA in complex with CA tubes in conjunction with molecular dynamics simulations identified a secondary CA binding site on CypA that allows a bridging interaction with two hexameric subunits of the assembled CA lattice, leading to capsid stabilization (Liu et al. in Nat Commun 7:10714, 2016).

**Results:**

We performed mutational analysis of residues that have been proposed to mediate CA binding at the secondary binding site on CypA (A25, K27, P29 and K30) and tested the effects of the amino acid substitutions using interaction assays and HIV-1 infection assays in cells. The binding of recombinant CypA to self-assembled CA tubes or native HIV-1 capsids was measured *in vitro* using a quantitative fluorescence microscopy binding assay revealing that affinity and stoichiometry of CypA to the CA lattice was not affected by the substitutions. To test for functionality of the CypA secondary CA-binding site in HIV-1 infection, mutant CypA proteins were expressed in cells in which endogenous CypA was deleted, and the effects on HIV-1 infection were assayed. In normal HeLa-P4 cells, infection with HIV-1 bearing the A92E substitution in CA is inhibited by endogenous CypA and was inhibited to the same extent by expression of CypA mutants in CypA-null HeLa-P4 cells. Expression of the mutant CypA proteins in CypA-null Jurkat cells restored their permissiveness to infection by wild type HIV-1.

**Conclusions:**

The amino acid changes at A25, K27, P29 and K30 did not affect the affinity of CypA for the CA lattice and did not impair CypA function in infection assays suggesting that these residues are not part of a secondary CA binding site on CypA.

**Electronic supplementary material:**

The online version of this article (10.1186/s12977-019-0471-4) contains supplementary material, which is available to authorized users.

## Background

The HIV-1 capsid is composed of about 1200–1500 copies of the viral CA protein arranged into a predominantly hexameric lattice with exactly 12 pentamers to form a closed conical shell that houses the viral RNA [1, 2]. After cell entry the capsid serves as a transport and reaction container that recruits a series of host co-factors to coordinate steps during the early stage of the HIV-1 life cycle [1, 3, 4] including reverse transcription [5, 6], cytoplasmic transport [7, 8], nuclear import [9, 10], and integration targeting of the viral DNA [9, 11–13]. Somewhere on its journey from the cell periphery into the nucleus, the capsid undergoes a disassembly process (termed uncoating) that releases the viral DNA for integration into the host chromosome. While there is controversy about the precise location and timing of uncoating in the cell, it is likely that capsid stability is also controlled by interaction of the capsid with proteins and small molecules that the virus collects from the producer cell and/or the host cell.

**Fig. 1:**
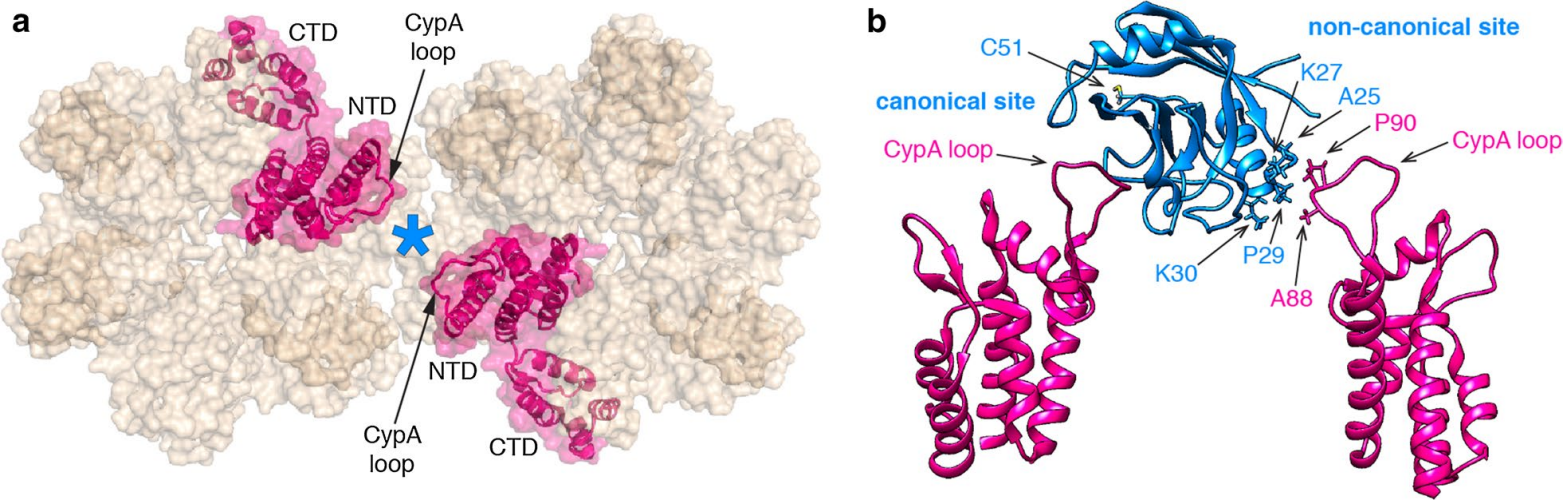
Proposed structural model of the interaction of CypA with the hexameric CA lattice. **a** Top view of two neighboring hexamers (PDB: 4XFX) showing the location of CypA (cyan asterisk) bridging between two CA molecules. The CA subunits that are bridged by binding of CypA are highlighted in magenta. **b** Side view of a structural model obtained from all-atom molecular dynamics (MD) simulations of the CypA-CA complex in a helical assembly (PDB: 5FJB). CypA (cyan) binds to the CypA loops of two CA subunits (magenta, only the N-terminal domains are shown) via its canonical binding site (left) and proposed non-canonical binding site (right), respectively. Residues involved in binding at the non-canonical binding site and the cysteine residue (C51) used for site-specific labeling are indicated

The peptidylprolyl isomerase cyclophilin A (CypA) is an abundant cytosolic protein that binds via its active site to the CypA binding loop located on the N-terminal domain of CA (CA-NTD). The CypA binding loop comprises CA residues 85 to 93 with G89 and P90 being the primary binding motif for CypA [14, 15] (Fig. 1b). The interaction of CypA molecules with multiple of these loops exposed on the outside of the capsid in the cytoplasm of the target cell modulates HIV infection in a cell type-specific manner [16, 17], possibly by regulating capsid stability and uncoating [18, 19]. For example, inhibition of this interaction in myeloid cells, either by using the competitive inhibitor cyclosporin A (CsA) or by mutation of residues in the CypA binding loop on CA, results in HIV-1 induction of host cell immune responses, which has been attributed to premature uncoating and release of viral DNA in the cytoplasm [20, 21]. The molecular mechanisms underlying modulation of infection are not fully understood but may involve dynamic allostery [22].

A recent cryoelectron microscopy structure of CypA in complex with self-assembled CA tubes revealed that CypA binds preferentially between CA hexamers along the direction of highest curvature [23]. The authors proposed that CypA bridges between the hexamers by binding to a CypA loop of one hexamer via the CypA active site (canonical site) and contacting a CypA loop of the second hexamer via a previously unidentified second (non-canonical) binding site (Fig. 1b). Molecular dynamics simulations suggested that the contact was mediated by A88, G89 and P90 from CA and A25, P29 and K30 from CypA [23]. The bridging interaction afforded by the second binding site is predicted to strengthen the interaction between CypA and the capsid as a result of avidity, stabilize the capsid in the cytoplasm and facilitate the effects of CypA in HIV-1 infection. Validation of this new site is therefore crucial for further dissecting the molecular mechanisms by which CypA affects the capsid during the early stages of the HIV-1 life cycle. Here we show that amino acid substitutions at CypA residues A25, K27, P29 and K30, which are proposed to mediate binding at the second site, did not affect the interaction of CypA with self-assembled CA tubes or native viral capsids *in vitro* nor the function of CypA in HIV-1 infection in HeLa-P4 or Jurkat cells. Our results suggest that these residues do not form a non-canonical capsid binding site.

## Materials and methods

### CypA expression and purification

The coding sequence for human CypA (wild type and mutants A25D, K27D, P29K and K30D) was amplified from the corresponding constructs for mammalian expression (see below) by PCR and cloned into a pET-21 vector using the XhoI and NdeI restriction sites to generate a construct for expression of CypA without purification tags. CypA was expressed in *E. coli* Rosetta (DE3) cultured in LB medium containing 100 μg/ml ampicillin and 32 μg/ml chloramphenicol at 37 °C with shaking to an optical density of 0.5. After induction with 1 mM IPTG and expression at 37 °C for 3 h, the cells were harvested by centrifugation, resuspended in cold purification buffer, whereby the buffer composition depended on the mutations because these result in changes of the isoelectric point of the protein: 25 mM HEPES, pH 7.6 [wild type CypA] or pH 7.2 [CypA A25D]; 50 mM MES, pH 6.8 [CypA K27D and CypA K30D]; 50 mM Bicine, pH 8.9 [CypA P29K]; each buffer was supplemented with 1 mM DTT, 0.02% NaN_3_, 1 mg/mL lysozyme and “Complete” protease inhibitor. The cells were then lysed by sonication on ice for 6 min (cycles of 15 s on/15 s off). The lysate was centrifuged at 43,000*g*, 4 °C for 1 h and the supernatant was filtered through a 0.22 μm membrane. All CypA variants were then partially purified by subtractive anion exchange chromatography using two HiTrap Q HP columns (2 × 5 mL connected in series, GE Healthcare) equilibrated with the corresponding purification buffer supplemented with 1 mM DTT and 0.02% NaN_3_. In subtractive anion exchange chromatography, the protein of interest elutes in the flow through during sample loading while contaminating proteins bind to the column and are thus removed. The next purification step differed for the CypA variants. Wild type CypA and mutants A25D and P29K were further purified by cation exchange chromatography. Pooled fractions containing CypA were adjusted in pH (pH 5.8 [wild type CypA]; pH 5.1 [CypA A25D]; pH 6.1 [CypA P29K]) using 1% v/v acetic acid prior to centrifugation at 43,000*g*, 4 °C for 1 h. The supernatant was injected onto HiTrap SP HP columns (2 × 5 mL connected in series, GE Healthcare) equilibrated with SP buffer, whereby the buffer composition depended on the mutation: 25 mM sodium phosphate, pH 5.8 [wild type CypA]; 50 mM sodium acetate, pH 5.1 [CypA A25D]; 50 mM BIS-TRIS, pH 6.1 [CypA P29K]; each buffer supplemented with 1 mM DTT, 0.02% NaN_3_. CypA was eluted using a linear gradient from 0 to 1 M NaCl in the corresponding SP buffer. CypA mutants K27D and K30D were further purified by gel filtration. Fractions containing CypA were combined, concentrated to yield a volume of approximately 600 μL and purified using a Superdex 200 Increase 10/300 GL column (GE Healthcare) equilibrated with gel filtration buffer (10 mM HEPES, pH 8, 2 mM EDTA, 100 mM NaCl). All CypA variants were dialyzed into CypA storage buffer (25 mM HEPES, pH 8, 1 mM DTT, 0.02% NaN_3_, 20% glycerol) prior to flash-freezing with liquid N2 and storage at − 80 °C.

### CypA fluorescence labeling

Recombinant CypA was labeled with a twofold excess of Alexa Fluor 647-C2-maleimide (Thermo Fisher, A20347) in PBS (pH 7.4) containing 0.1 mM TCEP at room temperature for 10 min in the dark before quenching with DTT. Unconjugated dye was removed using Zeba desalting columns (ThermoFisher) equilibrated with AF647-CypA storage buffer (50 mM Tris, pH 7.9, 1 mM DTT, 20% glycerol). Under these conditions, CypA is quantitatively labeled at residue C51. Wild type CypA labeled at C51 binds in surface plasmon resonance measurements to immobilized CA or CA hexamers with the same affinity as unlabeled CypA [24]. Labeled CypA was frozen in liquid nitrogen and stored at − 40 °C.

### CA expression and purification for assembly of tubes on surfaces

Expression and purification of CA mutants with engineered cysteine residues for cross-linking tubes (CA A14C/E45C) and for site-specific labeling (CA K158C) was carried out using published methods for the purification of CA [25].

### CA fluorescence labeling

CA K158C was labeled with Alexa Fluor 488-C5-maleimide at the engineered cysteine residue (Thermo Fisher) after assembly into CA tubes to avoid modification of native cysteine residues. CA K158C (7.36 mg/ml in 25 mM sodium phosphate buffer, pH 7, 0.02% NaN_3_, 2 mM DTT) was assembled for 15 min on ice after addition of solid NaCl to a final concentration of 2.5 M. The resulting CA tubes were collected by centrifugation (18,000*g*, 5 min) and resuspended in high salt buffer (50 mM Tris pH 8, 2.5 M NaCl, 0.1 mM TCEP, 0.02% NaN_3_). This centrifugation/resuspension process was repeated twice. A twofold molar excess of Alexa Fluor 488-C5-maleimide was then added to the resuspended CA K158C tubes, allowed to react for 1 min and unreacted dye was then quenched by the addition 2-mercaptoethanol to a final concentration of 25 mM. The labeled tubes were washed by four cycles of centrifugation and resuspension in high salt buffer as above. Finally, the tubes were collected by centrifugation, resuspended in buffer without salt to induce disassembly (50 mM Tris, pH 8, 0.1 mM TCEP, 0.02% NaN_3_). The solution was then centrifuged at 18,000*g*, 10 min, 4 °C to remove aggregates. The supernatant containing labeled CA was flash frozen in aliquots with liquid N_2_ and stored at − 40 °C in dark.

### NMR experiments

Uniform ^15^N/^13^C labeling of wild type CypA and CypA mutants A25K, P29D and P29K was performed by growth in modified minimal medium using ^15^NH_4_Cl and ^13^C_6_ glucose as the sole nitrogen and carbon sources, respectively. Expression, purification and uniform ^15^N/^13^C labeling of wild type CA-NTD for NMR was performed as described previously [26]. NMR experiments were conducted at 25 °C on ^15^N/^13^C wild type CA-NTD in the absence or presence of CypA (wild type or mutants), or ^15^N/^13^C CypA (wild type or mutants) in the absence and presence of wild type CA-NTD in NMR buffer (25 mM sodium phosphate buffer, pH 6.5, 1 mM DTT, 0.02% NaN_3_). A Bruker 600 MHz spectrometer equipped with a 5 mm triple resonance and z-axis gradient cryoprobe was used to obtain one-dimensional (1D) ^1^H and two-dimensional (2D) ^1^H-^15^N heteronuclear single quantum coherence (HSQC) spectra.

### Surface plasmon resonance (SPR) measurements

SPR measurements were performed on a Biacore S200 (GE Healthcare) as described previously [24]. Briefly, biotinylated CA K158C and biotinylated CA hexamer (assembled from CA A14C/E45C/W184A/M185A) were immobilized in separate flow channels of a CM5 sensor chip modified with streptavidin. Flow channels modified with streptavidin only were used as reference cells. CypA was exchanged into SPR running buffer (10 mM HEPES, pH 8, 0.005%Tween 20, 2 mM EDTA, 150 mM NaCl) using a Zeba desalting column and injected into the flow channels at a range of concentrations (1.1–72.8 μM) for 20 s followed by wash-out with SPR running buffer for 30 s at a flow speed of 100 μL/min while measuring the SPR response at a frequency of 40 Hz. SPR data were analyzed using the Biacore S200 Evaluation software and GraphPad Prism 7. Data for all background-corrected SPR traces are available in Additional file 2: Tables S1–S10.

### Fabrication of microfluidic flow cells

Glass coverslips were sonicated in ethanol for 30 min before sonication in 1 M NaOH for 30 min, and then rinsed with ddH2O and dried. PDMS devices with five flow channels (height 60 μm, width 800 μm) were prepared following standard soft lithography protocols. Both PDMS device and glass coverslip were exposed to an air plasma inside a plasma cleaner for 3 min before they were assembled into microfluidic flow cells. The microfluidic flow cell was then heated at 70 °C for at least 5 min to enhance bonding.

### Surface chemistry and CA tube assembly on surfaces

The surface of the glass cover slip forming the bottom of the microfluidic flow cell was coated with a co-polymer composed of poly-l-lysine (PLL) and biotinylated poly(ethylene glycol) (PEG) (Susos AG, PLL(20)-g[3.5]-PEG(2)/PEG(3.4)-biotin(20%)) by incubating each flow channel with a solution of PLL-PEG (1 mg/mL in PBS) for 30 min at room temperature before washing the channels with water and drying. The flow channels were then incubated with a solution of streptavidin (0.2 mg/mL) in blocking buffer (20 mM Tris, pH 7.5, 0.2 mg/mL BSA, 50 mM NaCl, 2 mM EDTA, 0.03% NaN_3_, 0.025% Tween 20) for 30 min at room temperature before rinsing the flow channels with 50 μL of washing buffer (50 mM Tris, pH 8). The flow channels were connected to inlet and outlet tubing and the entire flow cell mounted onto the microscope stage. The outlet tubing of the channel in use was connected to a syringe pump operated in withdraw mode at a flow speed of 100 μL/min unless otherwise specified. The flow channel was filled with an antibody mixture (20 μL, delivered at 100 μL/min) containing biotinylated anti-mouse IgG F(AB’)_2_ fragment (Jackson ImmunoResearch, 115-066-071; 1:800 dilution) and mouse anti-HIV-1 p24 monoclonal antibody (Advance Biotechnologies, 13-102-100; 1:800 dilution) and incubated for 1 min before rinsing with 100 μL of washing buffer. Then the flow cell was filled with CA tube assembly mixture (15 μL, delivered at 30 μL/min) containing CA-K158C-AF488 (4 μM), CA A14C/E45C (76 μM) in assembly buffer (50 mM Tris, pH 8, 500 mM NaCl; NaCl was added just before flowing the mixture into the flow channel) and incubated for 25 min to allow the growth of CA tubes on the modified surface.

### TIRF imaging of CypA binding to self-assembled CA tubes

The microfluidic flow cell was flushed with 100 μL of imaging buffer (50 mM Tris, pH 8, 150 mM NaCl, 2 mM trolox (Sigma-Aldrich) and 2.5 mM protocatechuic acid (Sigma-Aldrich)). CypA solutions (1–50 μM) were prepared with AF647-CypA (1 μM) and unlabeled CypA (added to make up the desired final concentration) in imaging buffer supplemented with protocatechuate-3,4-dioxygenase (0.25 U/mL, Sigma-Aldrich) shortly before injection into the flow channel. The CypA solution (30 μL) was flowed into the microfluidic channel and images were collected in both channels (CA and CypA) in four different fields of view. Subsequently the CypA solution was washed out with 100 μL of imaging buffer. This procedure was used to image CypA binding for a randomized sequence of different CypA concentrations in the same flow channel. TIRF images (5 frames per channel) were acquired with sequential excitation with a 491 nm laser (CA channel) and a 639 nm (CypA channel) with an exposure time of 10 ms using Andor iXon 888 EMCCD cameras. Images were analyzed using home-written image analysis software (https://github.com/lilbutsa/JIM-Immobilized-Microscopy-Suite). Regions of images containing capsid tubes were detected using the CA channel. The fluorescence intensity (sum of pixel values minus background intensity) for each tube was determined in each channel. Background fluorescence was determined from pixels surrounding the regions containing tubes.

The intensities of the two fluorophores were determined by single molecule photobleaching of labeled proteins adsorbed onto the surface of a coverslip. The absolute intensities of the two fluorophores were used to convert the fluorescence ratio of each region between the two channels (CypA:CA) to a molar binding ratio.

Binding curves were obtained by plotting the best fit molar binding ratio as a function of CypA concentration. The following equation was used for model fitting to obtain estimates for the dissociation constant and the maximum binding ratio: R(eq) = [CypA] × R(max) / ([CypA] + K_D_), where R(eq) is the CypA:CA ratio at equilibrium for a given CypA concentration, [CypA] is the concentration of CypA, R(max) is the CypA:CA ratio at saturation and K_D_ is the dissociation constant.

### TIRF imaging of CypA A25D binding to capsids in permeabilized viral particles

The protein–capsid interaction assay with viral particles immobilized inside the microfluidic flow channel was carried out as described previously. Briefly, HIV-1 particles were produced by transfecting HEK-293T cells with a mixture of pNL4.3-iGFP-ΔEnv and psPAX2 (1.4:1, mol/mol) using polyethylenimine (PEI Max) reagent (Polysciences, 9002-98-6). Supernatant containing viral particles was collected 48 h after transfection. The viral particles were biotinylated with EZ-Link Sulfo-NHS-LC-LC-Biotin (Thermo Scientific, 21338) and purified by size exclusion chromatography using a HiPrep 16/60 Sephacryl S-500 HR column (GE Healthcare) equilibrated with HBS (50 mM HEPES, pH 7.5, 100 mM NaCl). For imaging, viral particles were bound inside a microfluidic channel to the surface of a cover slip modified with PLL-PEG and streptavidin as described above. AF647-CypA A25D binding was imaged by time-lapse total internal reflection fluorescence microscopy (sequential acquisition, 491 nm and 561 nm laser, 20 ms exposure time, one frame per second) after injecting perfringolysin O (200 nM) in VLP imaging buffer (50 mM HEPES pH 7.0, 100 mM NaCl) supplemented with an oxygen quenching system to reduce photobleaching (2 mM trolox, 2.5 mM protocatechuic acid, 0.25 U mL-1 protocatechuate-3,4-dioxygenase). CypA solutions (0.1–20 mM) were prepared with AF647-CypA (1 μM) and unlabeled CypA (added to make up the desired final concentration) in VLP imaging buffer were assessed, whereby the concentration of the AF647-CypA A25D was used at a maximum concentration of 1 µM. Images were analysed with software written in MATLAB (The MathWorks, Inc.) as previously described [24].

### Generation of cells expressing mutant CypA proteins

Hela-P4 cells [27] were cultured in Dulbecco’s modification of Eagle’s medium (DMEM) containing 10% fetal bovine serum and antibiotics. Jurkat cells were cultured in RPMI1640 medium with the same supplements. The *PPIA* gene, encoding cyclophilin A, was disrupted in Hela-P4 cells by transduction with a pLentiCRISPRv2 lentiviral vector [28] encoding guide RNA sequence 5’-GTTCTTCGACATTGCCGTCGA-3’. The vector was packaged by cotransfecting 293T cells with psPAX2 (AddGene 12260) and pHCMV-G [29]. Transduced cells were selected in puromycin; single-cell clones were obtained by limiting dilution, expanded, and analyzed for CypA expression by immunoblotting. A cell clone lacking detectable CypA expression was reconstituted with wild type and mutant CypA proteins. For CypA reconstitution, pEF-IRES-bsd, a lentiviral vector containing the eEF1 promoter upstream of an IRES-Bsd cassette, was constructed from the EF.CMV.RFP lentiviral vector (Addgene plasmid # 17619; a gift from Linzhao Cheng) by substituting the IRES-Bsd cassette region, amplified by PCR from pMXs-IRES-Bsd (Cell Biolabs, cat. RTV-016), for the CMV-RFP region. Mutant CypA cDNAs with an upstream consensus Kozak sequence (CCACC) were created by site-directed mutagenesis via overlap PCR and inserted into the unique EcoRV site in pEF-IRES-Bsd. The orientation of the inserts was tested by restriction digestion, and the CypA cDNA inserts were confirmed by DNA sequencing. The resulting lentiviral vector constructs were packaged and transduced into the CypA-disrupted Hela-P4 cell clone. Transduced cells were selected with blasticidin, and the resulting populations were analyzed by immunoblotting for CypA expression. For Jurkat cell lines, Jurkat *PPIA *−/− cells [30] obtained from the NIH AIDS Reagent Program, were reconstituted by transduction with EF-CypA-IRES-Bsd vectors. Blasticidin-resistant populations were selected and used in HIV-1 infection assays.

### Infection assays

Cells were assayed for permissiveness to HIV-1 infection by flow cytometric analysis of infected cells expressing GFP following inoculation with HIV-1 reporter viruses encoding GFP. VSV-G-pseudotyped reporter particles were produced by co-transfection of 293T cells with pHIV-GFP [31] and pHCMV-G. Culture supernatants were harvested and clarified by centrifugation, assayed for p24 by ELISA, and frozen in aliquots at − 80 °C. Jurkat cells were suspended at 200,000 per ml and distributed into 96-well plates (100 μL per well), and dilutions of the reporter viruses were added (100 μL volumes) with polybrene at a final concentration of 5 μg/ml in the presence and absence of cyclosporin A (5 μM). Virus stocks were diluted to achieve infection levels from 1-30% to ensure accurate quantification. Two days after inoculation, cells were fixed overnight with 2% paraformaldehyde. For Hela-P4 infection assays, 10,000 cells were seeded overnight in wells of 96-well plates (100 μL volumes). Monolayers were inoculated with viruses in the presence of polybrene for 48 h, after which cells were detached with trypsin and fixed in paraformaldehyde. Fixed cell populations were analyzed for GFP expression with an Accuri C6 flow cytometer. The extent of infection was expressed as %GFP + cells. Infection assays were performed in duplicate and the average values determined.

### Immunoblotting

Cells were detached with trypsin and complete medium was added. Cells were pelleted, washed once in PBS, and cytoplasmic extracts prepared by lysis in 100 mM Tris-HCl pH 8.0, 100 mM NaCl, 0.5% NP-40. Protein concentrations were determined by the bicinchronic acid method (Pierce). Normalized quantities of total protein were subjected to SDS-PAGE on 4–20% polyacrylamide gradient gels (GenScript). After separation, the proteins were transferred to nitrocellulose membranes (Perkin Elmer) and probed with polyclonal antibody to cyclophilin A (Millipore, Cat. #07-313) at a 1:2000 dilution and antibody to GAPDH (Thermo Fisher, Cat.#A21994; 5 μg/ml). Antibody complexes were detected with the appropriate IR dye-conjugated secondary antibodies (LI-COR Biosciences) and detected by scanning with a LI-COR Odyssey imaging system. Bands were quantified using the Odyssey imaging software.

## Results

We generated single site CypA mutants (A25D, K27D, P29K and K30D) at four residues located at the secondary CA contact site (Fig. 1b) and tested their ability to disrupt CA binding biochemically and affect HIV-1 infection in cells. Additional mutants at these residues (A25K, K27A and P29D) were also tested in the infection assays. Introduction of a charged amino acid (lysine or aspartic acid) at residues A25 and P29 was designed to disrupt possible hydrophobic interactions with CA. The mutation at P29 (located at the N-terminal side of an α-helix) may also lead to localised main-chain displacement. Mutating K27 and K30 to an aspartic acid residue replaces a hydrogen bond donor with a hydrogen bond acceptor. In addition, these mutations, as well as K27A, change the steric configuration at these sites. K30D would also be expected to locally destabilise the structure by breaking the interaction of K30 with E83 that is apparent in the crystal structure of wild type CypA [32].

To test for avidity arising from the bridging binding mode of CypA with CA lattices (Fig. 1a), we compared the affinity of recombinant wild type and mutant CypA (A25D, K27D, P29K and K30D) for unassembled CA, cross-linked CA hexamers and self-assembled CA tubes. Biophysical characterization of purified proteins by differential scanning fluorimetry and circular dichroism (Additional file 1: Figure S1) showed that mutants A25D, K27D and P29K were similar to wild type CypA with respect to melting point and secondary structure content, whereas CypA K30D was destabilized, possibly as a result of removing the interaction of K30 with E83 as discussed above. 1D ^1^H spectra of CypA A25K and P29D mutants (not shown) and 2D ^1^H-^15^N HSQC NMR spectrum of ^15^N/^13^C-labeled CypA P29K mutant (Additional file 1: Figure S2B) reveal that the structures of the mutants are essentially the same as for wild type protein (Additional file 1: Figure S2A), whereby perturbations were confined to the mutated site and neighbouring residues while the active site was intact. Moreover, the binding property of CypA P29K (Additional file 1: Figure S2D) to CA-NTD, studied by ^1^H-^15^N HSQC, was the same as for wild type protein (Additional file 1: Figure S2C), i.e. the same set of CypA residues were perturbed by CA-NTD. Similarly, the ^1^H-^15^N HSQC spectral perturbations of ^15^N/^13^C-labeled CA-NTD upon adding unlabeled CypA P29K, P29D and A25K were similar (not shown). In summary, the binding data by NMR indicate that these CypA residues are not involved in the interaction with monomeric CA in solution.

**Fig. 2:**
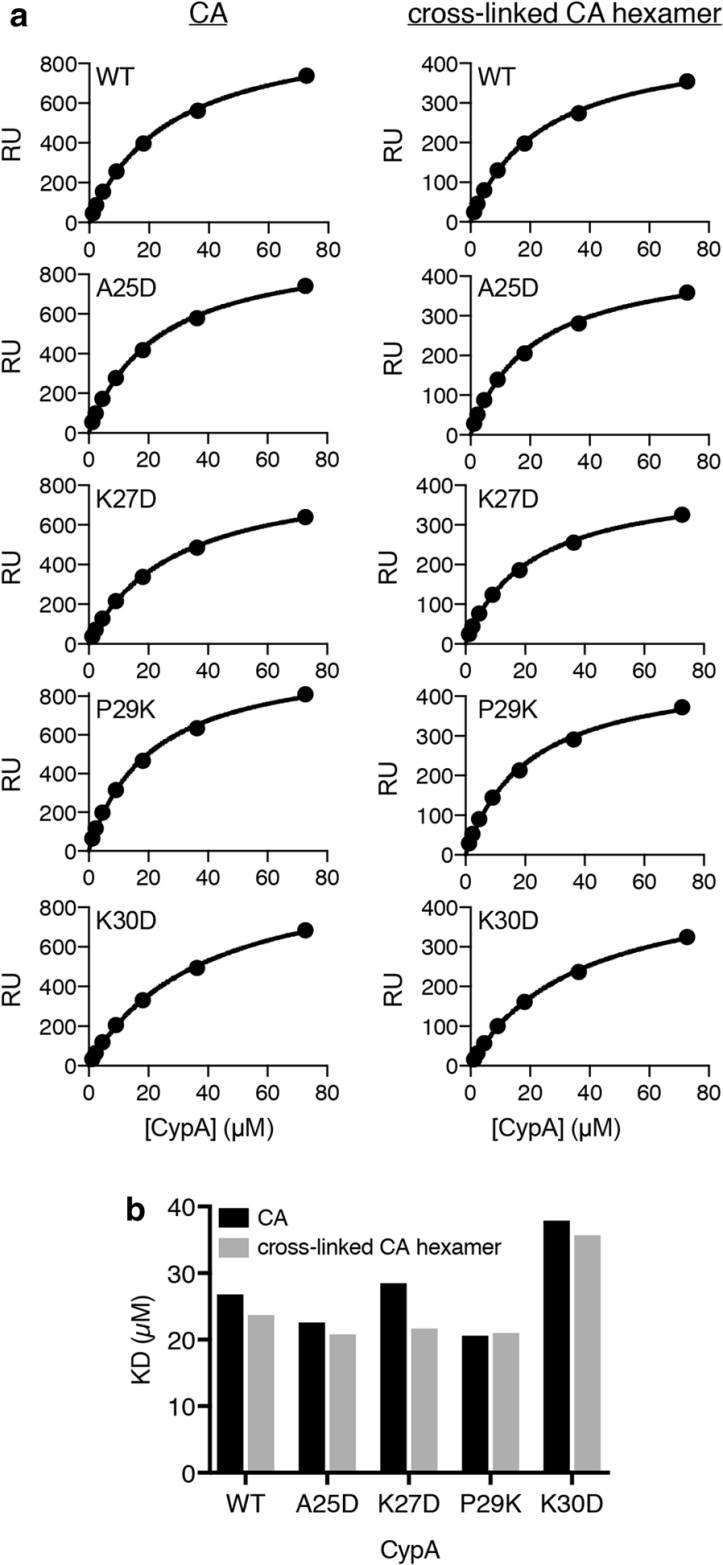
Binding affinity of wild type and mutant CypA for CA and cross-linked CA hexamers. Equilibrium analysis of surface plasmon resonance (SPR) curves of CypA binding on surfaces modified with CA K158C (reacted with a maleimide derivative of biotin) or cross-linked CA A14C/E45C/W184A/M185A hexamers (reacted with an NHS ester derivative of biotin). The SPR curves are shown in Additional file 1: Figure S2. **a** SPR response at equilibrium as a function of CypA concentration and fit of a binding model to the data (black line) to obtain the dissociation constant of the CypA interaction. **b** Equilibrium dissociation constants (K_D_) determined for wild type CypA and mutant interacting with CA and CA hexamers from the single experiments shown in A

We first used surface plasmon resonance (SPR) to determine the dissociation constant (K_D_) for all CypA variants interacting with CA immobilized on the surface of the SPR chip (Fig. 2 and Table 1, Additional file 1: Figure S3). The K_D_ for the interaction of wild type CypA with unassembled CA (K_D_ ~ 27 μM) was in the range of values determined previously by a range of techniques (7 μM [9] or 16 ± 4 μM [14] measured by isothermal titration microcalorimetry; 30 μM [33] or 21.8 μM [24] measured by SPR). As expected, the affinity of the interaction was essentially the same when using cross-linked CA hexamers (Fig. 2) because the bridging binding mode is not available within a single hexamer but only available between hexamers in a lattice (Fig. 1a). The K_D_ values for mutants A25D, K27D and P29K interacting with unassembled or hexameric CA were similar to the values observed for wild type CypA (Table 1) confirming that these mutations did not affect the integrity of the active site. The destabilized mutant K30D showed only a minor defect in binding via the active site with a 1.5—twofold increase in K_D_ compared to the other variants.

**Table 1:** Dissociation constants (K_D_/μM) for the CypA–CA interaction determined by SPR for CA and cross-linked CA hexamers (single experiments) and by fluorescence imaging for cross-linked CA tubes and native capsids (mean ± standard deviation)

	CA	CA hexamer	CA tube	Native capsid
WT	26.8	23.7	14.5 ± 5	10.7 ± 0.7
A25D	22.6	20.8	17.2 ± 4.8	8.7 ± 2.1
K27D	28.5	21.7	17.7 ± 6.7	n.d.
P29K	20.6	21	16.4 ± 3.8	n.d.
K30D	37.9	35.7	17 ± 9.2	n.d.

*n.d.* Not determined

**Fig. 3:**
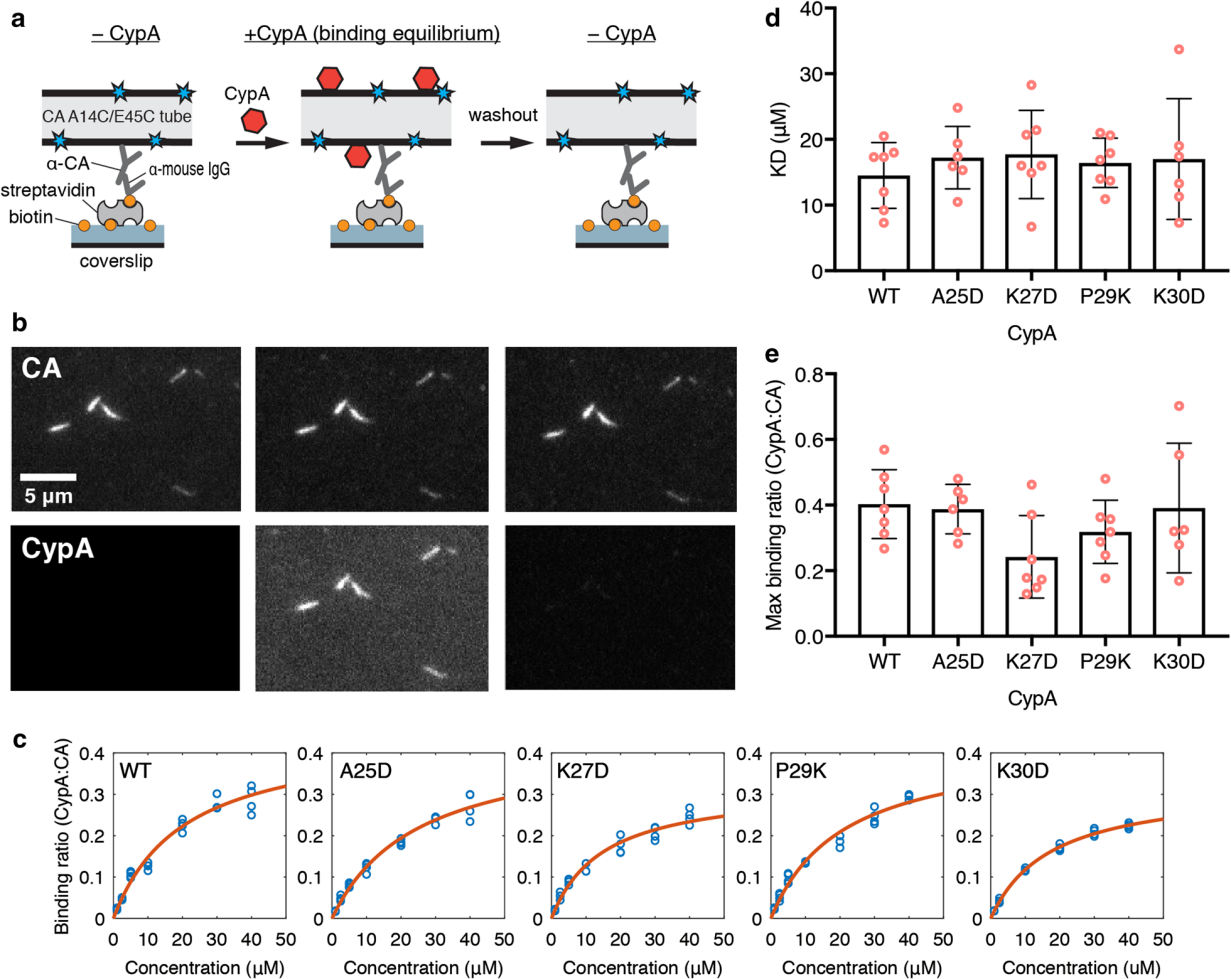
TIRF microscopy analysis of wild type and mutant CypA binding to cross-linked CA tubes. **a** Schematic diagram of the TIRF microscopy binding assay using cross-linked CA tubes assembled inside the flow cell from CA A14C/E45C and CA K158C-AF488 (20:1, mol/mol) and immobilized on the surface via an antibody. AF647-CypA injected into the flow cell co-localises with the tubes upon binding. **b** TIRF images of the capsid channel (top) and CypA channel (bottom) before CypA addition (left), in the presence of CypA (middle), and after CypA washout (right). **c** Representative equilibrium binding curves of the interaction of wild type or mutant CypA and CA tubes determined by TIRF microscopy. Each symbol represents the mean ratio determined for all tubes in a field of view. **d, e** Dissociation constants (K_D_) (D) and maximum CypA:CA molar binding ratios (E) determined from fits of equilibrium binding curves for wild type and mutant CypA. Each symbol represents an independent experiment. **d** The K_D_ values were estimated to be 14.5 ± 5 μM (WT), 17.2 ± 4.8 μM (A25D), 17.7 ± 6.7 μM (K27D), 16.4 ± 3.8 μM (P29K) and 17 ± 9.2 μM (K30D) (mean ± SD, n ≥ 6). *P* ≥ 0.86 between CypA WT and mutants (one-way ANOVA). **e** Maximum binding ratios of CypA binding with CA tubes (molar ratio of CypA:CA when saturated) were estimated at 0.40 ± 0.11 (WT), 0.39 ± 0.08 (A25D), 0.24 ± 0.13 (K27D), 0.32 ± 0.10 (P29K) and 0.39 ± 0.20 (K30D) (mean ± SD, n ≥ 6). *P* ≥ 0.15 between CypA WT and mutants (one-way ANOVA)

Next, we investigated the interaction of wild type and mutant CypA with the CA lattice. Capsid binding of host proteins is commonly investigated using co-sedimentation with CA (or CA-NC) tubes as surrogates for the native capsid lattice. Recombinant CA self-assembles into tubular lattices made up of CA hexamers in the presence of high salt in the buffer. These tubes can be stabilized to prevent disassembly at physiological salt concentrations by using a double cysteine mutant (CA A14C/E45C) that forms disulfide cross-links between adjacent CA subunits in each hexamer. Here we used a quantitative fluorescence imaging assay to measure the affinity and stoichiometry of CypA variants binding to cross-linked CA tubes, as illustrated in Fig. 3a. CA tubes were grown inside a microfluidic flow-channel in a high salt solution by co-assembly of CA A14C/E45C and CA K158C modified with AlexaFluor 488 (for fluorescence labeling of the tubes) and immobilized onto the modified surface of the glass cover slip via an antibody directed against the CypA loop of CA. We then measured binding and dissociation of CypA labeled at Cys51 with Alexa Fluor 647 (Fig. 1b) flowed into the channel at a range of concentrations and then washed out while imaging the fluorescence signals of the CA tubes and bound CypA by total internal reflection fluorescence (TIRF) microscopy. CA tubes were detected as lines in the fluorescence image (Fig. 3b, top). The signal of AF647-CypA co-localized with the tubes and rapidly reached a constant level after injection into the flow channel (Fig. 3b, bottom). Wash-out with buffer led to complete disappearance of the AF647-CypA signal indicating rapid dissociation (Fig. 3b, bottom). The ratio of the fluorescence intensity for each tube in the CA channel to the corresponding intensity in the CypA channel was converted to a molar binding ratio (see Materials and Methods). Analysis of the binding curves measured for tubes in multiple fields of view at a range of CypA concentrations with an equilibrium binding model (Fig. 3c) then yielded an estimate of K_D_. There was no statistically significant difference in the affinity of the interaction between CypA and CA tubes for wild type (K_D_ ~ 15 μM) and mutant CypA (K_D_ ~ 16–18 μM), including the destabilized mutant K30D (Fig. 3d and Table 1). The binding curves further revealed that CypA binding to the CA lattice saturated when ~ 40% of CypA loops on the lattice are occupied with a CypA molecule irrespective of mutation (Fig. 3e). This binding ratio is close to the maximum ratio that is expected when taking steric hindrance between CypA molecules interacting with neighboring CypA loops on the lattice into account [23]. We conclude that substitutions of residues at the proposed secondary binding site on CypA do not affect the affinity or stoichiometry of CypA binding to the hexameric lattice of cross-linked CA tubes.

**Fig. 4:**
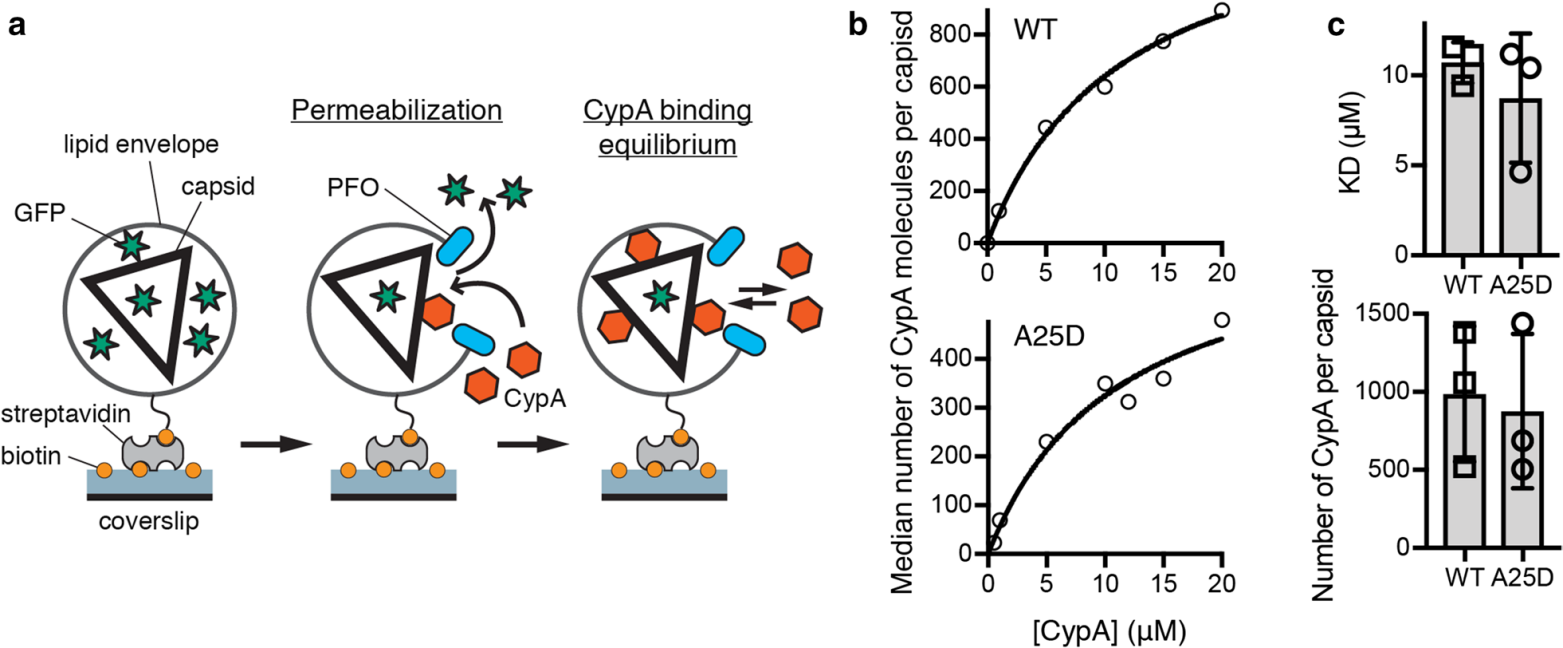
CypA A25D binds with the same affinity as wild-type CypA to authentic HIV-1 capsids. **a** Schematic diagram of the TIRF assay for measuring CypA binding to the intact capsid. **b** Representative equilibrium binding curves obtained for wild type (top) and A25D (bottom) by plotting the mean number of CypA molecules bound per capsid at equilibrium as a function of CypA concentration. The fit of an equilibrium binding model (black line) gave estimates for K_D_ and number of molecules bound at saturation; wild type: K_D_ = 11.6 μM, number of molecules = 1381; A25D: K_D_ = 11.2 μM, number of molecules = 688. **c** K_D_ (top graph) and mean number of CypA molecules bound per capsid at saturation (corresponding to the number of CypA loops that can be occupied simultaneously) (bottom graph) for the interaction of wild type CypA and CypA A25D with the capsid determined in independent experiments using different virus preparations; each symbol represents an independent experiment. The error bars represent standard deviations

Finally, we chose CypA A25D as one of the secondary site mutants without folding defects to determine whether its interaction with the wild type CA lattice of authentic viral cores was impaired compared to wild type CypA. The binding affinity and the number of CypA loops that can be occupied simultaneously on the assembled capsid were determined using our recently developed *in vitro* single-molecule fluorescence imaging assay (Fig. 4a) [24]. Viral particles containing GFP as a solution phase marker were immobilized on the glass surface of a cover slip and permeabilized with a pore-forming protein to allow access of CypA to the capsid. Permeabilized virions with intact capsids retain encapsidated GFP molecules and were detected as diffraction-limited spots in the GFP channel. The fluorescence intensity of AF647-CypA injected into the flow channel was then monitored by TIRF microscopy at each location corresponding to an intact capsid, reaching a rapid equilibrium level. The AF647-CypA fluorescence intensity was then converted to the number of CypA molecules bound using a calibration value obtained by single molecule photobleaching. Representative equilibrium binding curves obtained by plotting the mean number of molecules per capsid as a function of CypA concentration are shown in Fig. 4b for wild type (top) and mutant (bottom). The K_D_ of CypA A25D binding to intact capsids (8.7 ± 2.1 μM) was not significantly different from the K_D_ measured for wild type CypA in the same system (10.7 ± 0.7 μM) (Fig. 4c). Likewise, the number of CypA loops occupied at saturation, as determined from the fit of the equilibrium binding curve, was not significantly different for mutant (877 ± 286) and wild type (987 ± 251) CypA (Fig. 4d). Taken together our observations with cross-linked tubes and authentic capsids suggest that mutation of secondary site residues does not affect binding of CypA to the CA lattice *in vitro*.

To test for functionality of the CypA secondary CA-binding site in HIV-1 infection, we expressed mutant CypA proteins in cells in which endogenous CypA was deleted and assayed the effects of secondary site mutations on HIV-1 infection. In the following sections we present results from infection assays in (1) HeLa-P4 cells (using HIV-1 with CA A92E mutation) and (2) Jurkat cells.

**Fig. 5:**
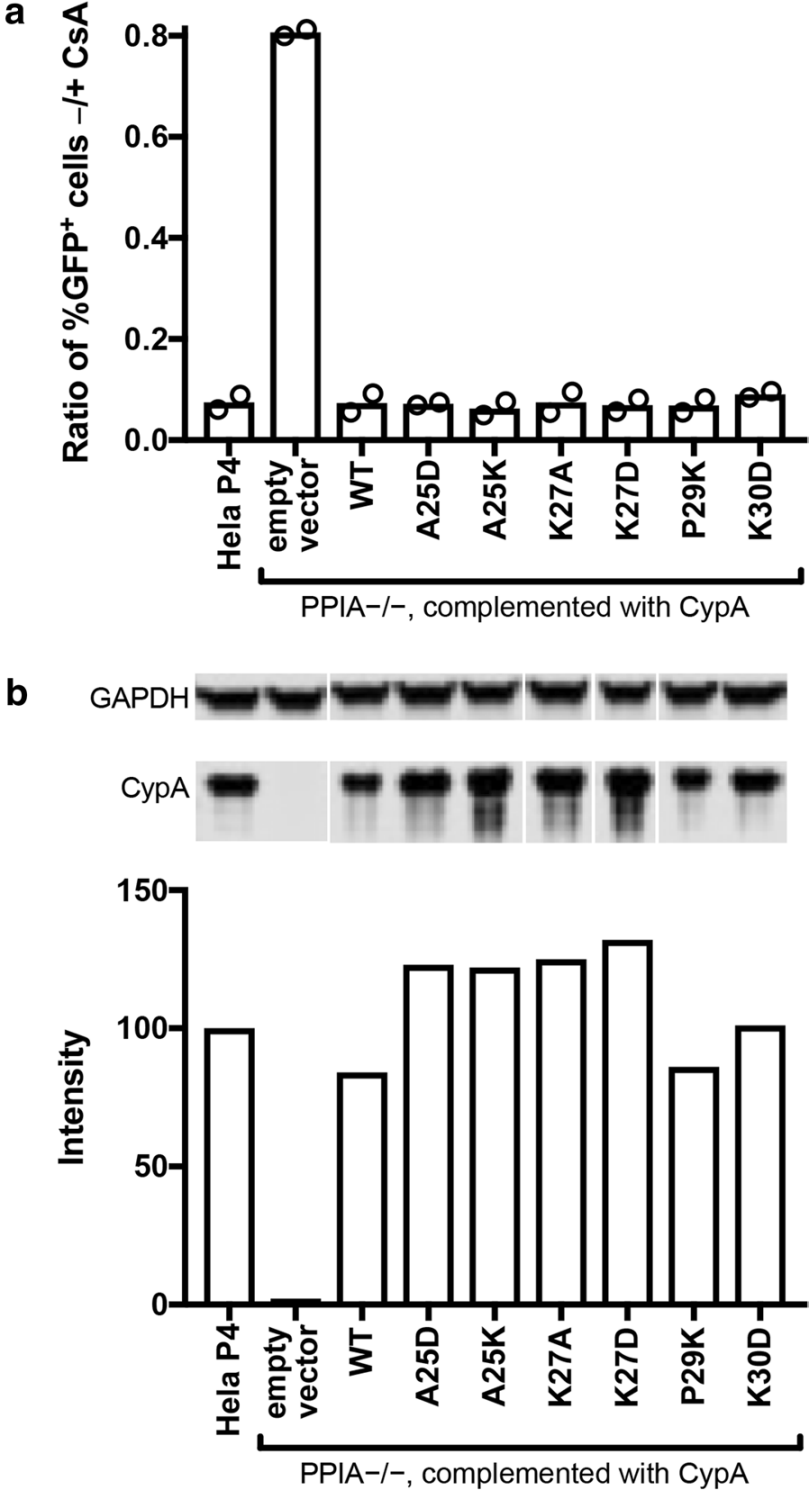
Functional analysis of CypA mutants in Hela-P4 cells. The *PPIA* gene was disrupted in Hela-P4 cells with CRISPR technology. The cloned cell line was transduced with a lentiviral vector encoding the indicated CypA mutant cDNAs. **a** Analysis of the extent of HIV-1 infection of the indicated cell lines by the A92E CA mutant in the presence and absence of CsA. A higher ratio indicates less enhancement of infection by CsA. Shown are the results of two independent determinations. **b** Quantitative analysis of CypA expression by immunoblotting relative to wild type HeLa-P4 cells. All samples were separated on the same gel and CypA and GAPDH were stained on the same blot membrane. CypA signals were normalized by the corresponding GAPDH signals

The HIV-1 capsid escape mutant A92E arises during passage of HIV-1 in HeLa-P4 cells during CypA inhibition [34] and is poorly infective in cells expressing high CypA levels such that its replication becomes dependent on the presence of the CypA inhibitor cyclosporin A (CsA) [35]. In HeLa-P4 cells, which express high levels of endogenous CypA, infectivity of an HIV-1 A92E reporter virus in the absence of CsA was less than 10% of the infectivity in the presence of CsA (− CsA/+ CsA infection ratio < 0.1, Fig. 5a). As expected, removal of CypA expression by disruption of the *PPIA* gene with CRISPR technology resulted in efficient HIV-1 A92E infection without the need for CypA inhibition (− CsA/+ CsA infection ratio > 0.8, Fig. 5a). Wild type CypA and CypA mutants A25D, A25K, K27A, K27D, P29K and K30D could be expressed in the CypA-null cells at near or above endogenous levels (Fig. 5b) using lentiviral vectors, which inhibited HIV-1 A92E infection and thus restored the CsA dependence to the same extent for all variants (− CsA/+ CsA infection ratio < 0.1, Fig. 5a).

**Fig. 6:**
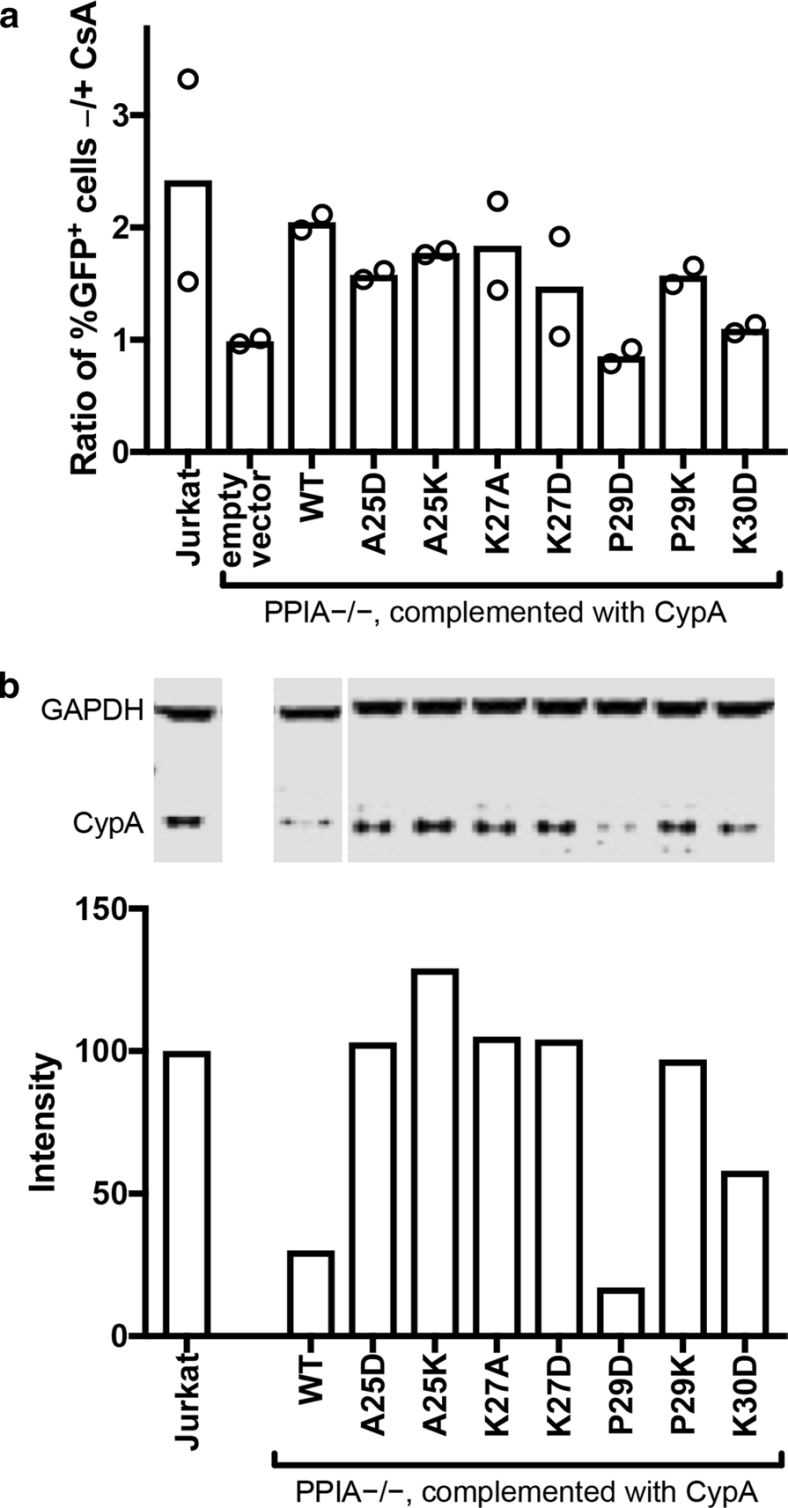
Functional analysis of selected CypA mutants in Jurkat cells. Jurkat *PPIA *−/− cells were transduced with lent viral vectors encoding the indicated wild type (WT) and mutant CypA proteins containing the indicated amino acid substitutions. **a** Analysis of the extent of infection of the indicated cell lines by HIV-1 in the presence and absence of cyclosporin A (CsA). The results shown are from two independent assays. **b** Analysis of CypA expression by immunoblotting relative to wild type Jurkat cells. Signals were normalized by the corresponding GAPDH signals from the same blot. All samples were separated on the same gel and CypA and GAPDH were stained on the same blot membrane. CypA signals were normalized by the corresponding GAPDH signals

CypA enhances HIV-1 infection in Jurkat cells [30, 36] and accordingly we found that infection of Jurkat cells with an HIV-1 reporter virus expressing GFP was about twice that in the absence of CsA vs. in the presence of CsA (Fig. 6a). As expected, HIV-1 infection was the same in the absence and presence of CsA in CypA-null Jurkat cells (− CsA/+ CsA infection ratio = 1, Fig. 6a), confirming that the antiviral effect of CsA is via inhibition of CypA. Expression of CypA proteins with mutations in secondary site residues A25 and K27 in CypA-null Jurkat cells restored their permissiveness to infection by wild type HIV-1 close to the level observed for wild type CypA, while the destabilized mutant CypA K30D showed partial enhancement of infectivity (see also Additional file 1: Figure S4). Expression of CypA P29K also enhanced HIV-1 infection close to wild-type levels, while CypA P29D did not restore permissiveness to infection, possibly because the expression level of this mutant was too low in CypA-null Jurkat cells (approximately half of the expression level of wild type CypA in CypA-null Jurkat cells, Fig. 6b). In additional experiments, expression of the P29D and P29K mutants was undetectable, and the phenotype of the corresponding cell lines was identical to that of the CypA-null cells (Additional file 1: Figure S4). Taken together these results suggest that the second capsid binding site on CypA is not functional in the infection assays we employed or was not affected by the amino acid changes tested thus far.

## Discussion and conclusion

Our mutational analysis of CypA residues at the proposed second CA binding interface revealed that introduction of a charged residue at A25 or P29, or charge reversal at K27 or K30, had no effect on: (1) CypA binding affinity or stoichiometry with the CA lattice *in vitro*; (2) the ability of the protein to inhibit infection of HeLa-P4 cells by a HIV-1 CA escape mutant that is dependent on CsA for replication; and (3) the ability of CypA to enhance HIV-1 infection of Jurkat cells. On the basis of these observations we conclude that the residues investigated here do not constitute a secondary binding interface for CA. Mutational analysis of CA was not carried out because the same residues (A88, G89, P90) implicated in binding to the non-canonical site of CypA also interact with the active (canonical) site of CypA, where they are buried into a hydrophobic groove [15].

Our *in*-*vitro* measurements of CypA–CA interactions utilized primarily self-assembled CA A14C/E45C tubes, which are similar in structure to the wild type CA tubes used previously to determine the cryoEM structure of the complex [23]. The CypA:CA stoichiometry of ~ 0.3–0.4 (corresponding to about two CypA molecules per hexamer) we obtained for all CypA variants is consistent with the computationally generated binding models reported for the tubular complex [23]. The additional interaction via the second site for CypA molecules along the most highly curved direction of the tube (corresponding to one CypA molecule per CA hexamer) would be expected to strengthen CypA–CA lattice interaction through avidity. Disabling the second site, e.g. via mutation, should weaken the interaction of mutant CypA with the tube compared to the wild type protein, which was not observed in our assay. The cross-linked hexamer used here to stabilise tubes at physiological salt concentrations structurally resembles the native hexamer [37] but cross-linking could in principle affect interactions with the lattice, e.g. as a result of lattice rigidification. As an additional test, we used a new fluorescence imaging assay that can quantify the number of CypA molecules interacting with native viral capsids to compare one of the mutants (A25D) to the wild type protein, but we observed no differences in affinity.

Similarly, our HIV-1 infection assays in HeLa-P4 and Jurkat cells revealed no functional differences between wild type CypA and the second site mutants, suggesting that the site was misidentified or does not play a role in the CypA use by HIV-1 capsid during the early life cycle.

The mutational analysis of residues conducted here does not rule out the existence of a secondary CA binding site and future structural studies of the complex at higher resolution may identify a different set of CypA residues that could constitute such a site. Our observation that the binding of wild type CypA to the CA lattice is only modestly stronger with the CA lattice than with unassembled CA (up to twofold reduction in K_D_ for CA tubes and up to threefold reduction in K_D_ for native capsids) suggests that binding at the second site, if present, is considerably weaker than at the active site and/or only accessible at few locations on the capsid lattice.

## Additional files

**Additional file 1.** Biochemical and structural characterization of CypA mutants, SPR sensorgrams, additional infection assays in Jurkat cells expressing CypA mutants.

**Additional file 2.** Tables of SPR sensorgram data.

## Abbreviations

CsA: cyclosporine A
CypA: cyclophilin A
NMR: nuclear magnetic resonance
NTD: N-terminal domain
SPR: surface plasmon resonance
TIRF: total internal reflection fluorescence
