# Supplementary material for: Functional analysis of the secondary HIV-1 capsid binding site in the host protein cyclophilin A
Source: Retrovirology. 2019 Apr 4;16:10. doi: 10.1186/s12977-019-0471-4 (PMC6449974; doi:10.1186/s12977-019-0471-4)
Supplement: Supplementary file 1 — Additional file 1. Biochemical and structural characterization of CypA mutants, SPR sensorgrams, additional infection assays in Jurkat cells expressing CypA mutants. [file 12977_2019_471_MOESM1_ESM.pdf]

Supplementary information

Functional analysis of the secondary HIV-1 capsid binding site in the host protein cyclophilin A

Wang Peng, Jiong Shi, Chantal Márquez, Derrick Lau, James Walsh, K. M. Rifat Faysal, Chan H. Byeon, In-Ja L. Byeon, Christopher Aiken, Till Böcking

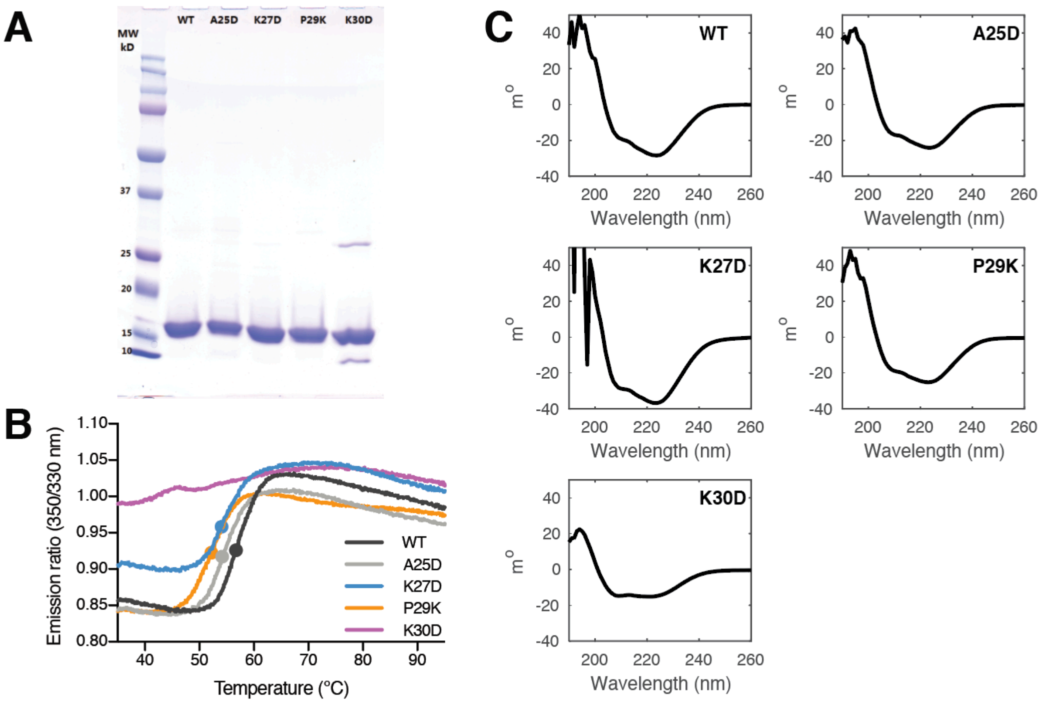

Supplementary Figure S1. Biochemical and structural characterization of recombinant CypA. A. SDS PAGE analysis with Coomassie staining of purified wild type and mutant CypA expressed in bacteria. **B.** Melting curves of wild type and mutant CypA measured by differential scanning fluorimetry. Melting temperatures (indicated by the dots) could be estimated for wild type CypA (56.8 °C) and mutants A25D (54.2°C), K27D (54.1°C) and P29K (52.2°C). The melting curve for CypA K30D showed a high fluorescence emission ratio across the entire temperature range (35–95 °C) indicating that this mutant is not properly folded. **C.** Circular dichroism spectra of wild type and mutant CypA.

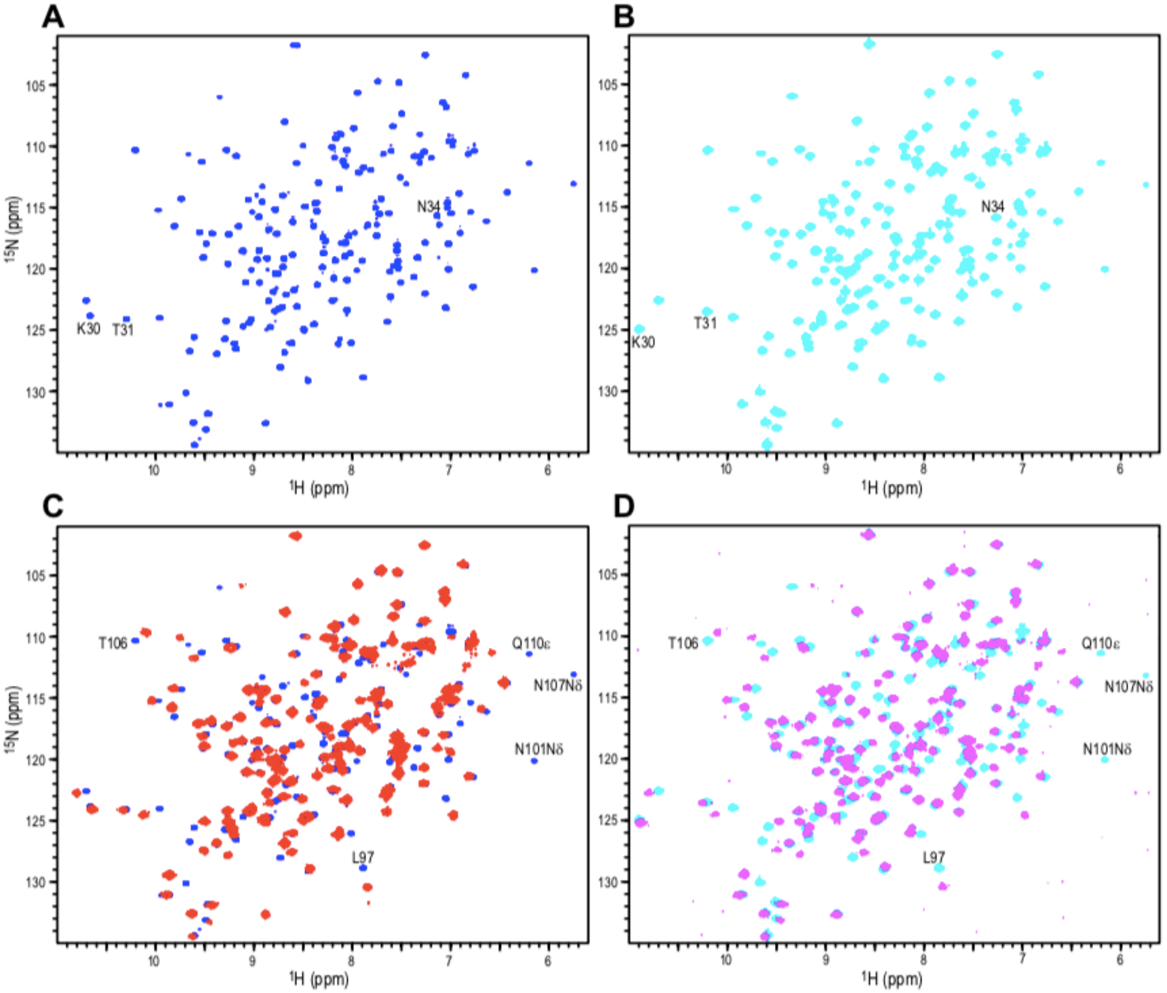

Supplementary Figure S2. ^{1}H-^{15}N HSQC spectra show that wild type CypA and CypA P29K have essentially the same structure and CA-NTD-binding property.

A. ^{1}H-^{15}N HSQC spectrum of $^{15}N/^{13}C$ wild type CypA.

B. ^{1}H-^{15}N HSQC spectrum of $^{15}N/^{13}C$ CypA P29K.

C. Superimposed ^{1}H-^{15}N HSQC spectra of $^{15}N/^{13}C$ wild type CypA alone (blue) and with 2-fold molar excess of CA-NTD (red).

D. Superimposed ^{1}H-^{15}N HSQC spectra of $^{15}N/^{13}C$ CypA P29K alone (cyan) and with 2-fold molar excess of CA-NTD (magenta).

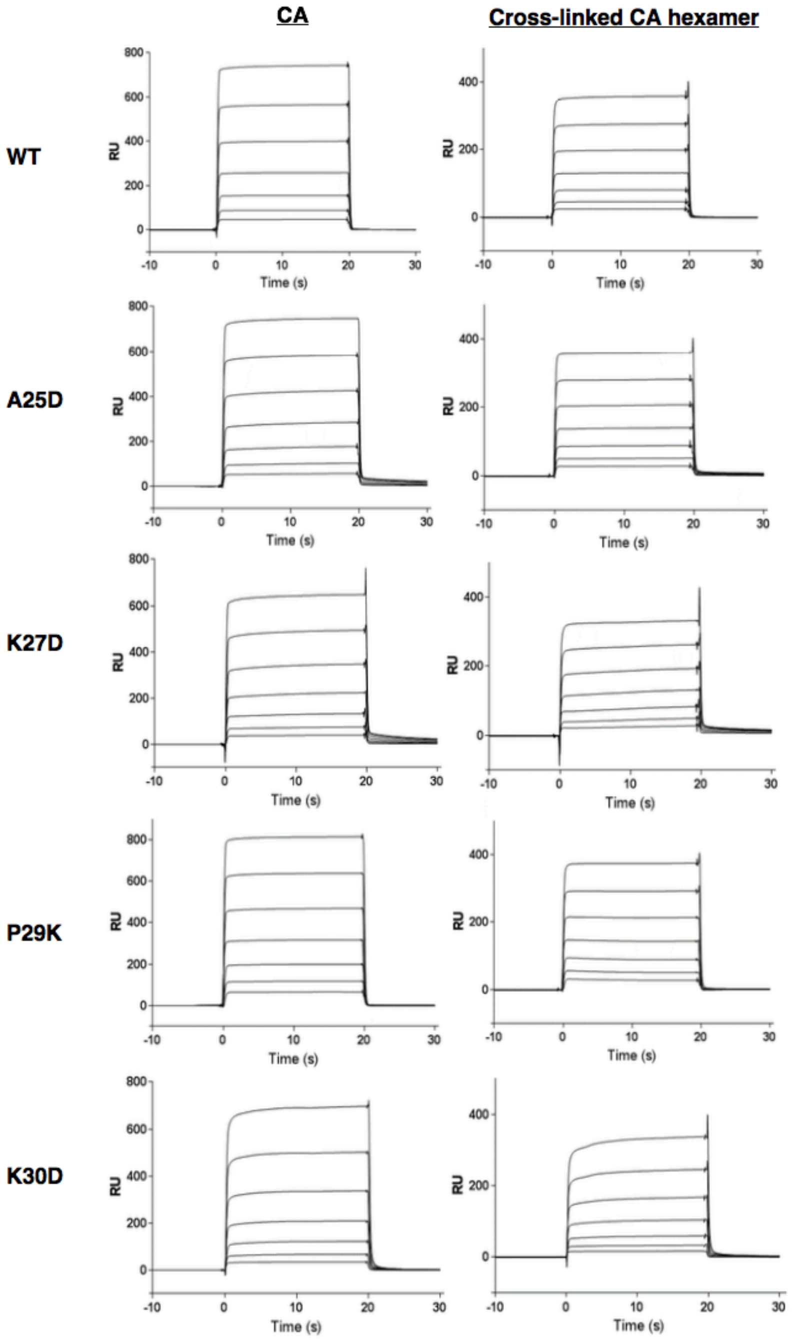

Supplementary Figure S3. Surface plasmon resonance spectroscopy traces of wild type and mutant CypA interacting with CA and cross-linked CA hexamers. CypA WT, A25D, K27D, P29K and K30D at a range of concentrations (1.1, 2.3, 4.5, 9.1, 18.2, 36.4 and 72.8 µM corresponding to curves from bottom to top) were flowed through SPR chip flow cells for 20s followed by wash-out while SPR response were recorded. SPR responses in CA monomer/hexamer flow cells were corrected from reference responses.

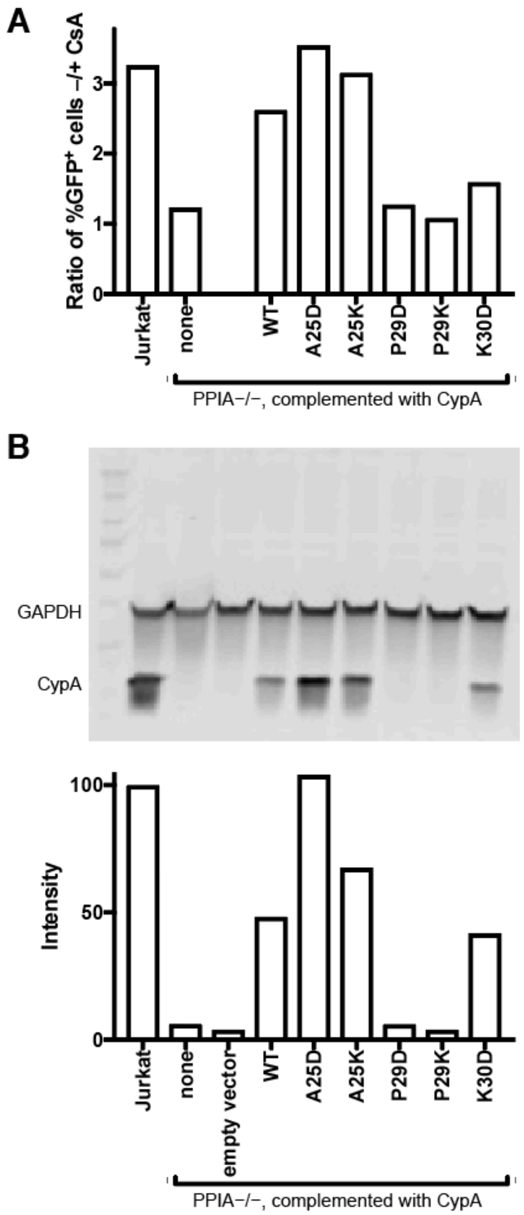

Supplementary Figure S4. Additional functional analysis of selected CypA mutants in Jurkat cells. Jurkat *PPIA−/−* cells were transduced with lentiviral vectors encoding the indicated wild type (WT) and mutant CypA proteins containing the indicated amino acid substitutions. (A) Analysis of the extent of infection of the indicated cell lines by HIV-1 in the presence and absence of cyclosporin A (CsA). The results shown are from one independent assay. (B) Analysis of CypA expression by immunoblotting relative to wild type Jurkat cells. Signals were normalized by the corresponding GAPDH signals from the same blot. All samples were separated on the same gel and CypA and GAPDH were stained on the same blot membrane. CypA signals were normalized by the corresponding GAPDH signals.
